# Paving the way while playing catch up: mitochondrial genetics in African ancestry primary open-angle glaucoma

**DOI:** 10.3389/fopht.2023.1267119

**Published:** 2023-10-12

**Authors:** Grace Kuang, Rebecca Salowe, Joan O’Brien

**Affiliations:** Penn Medicine Center for Genetics in Complex Disease, Scheie Eye Institute, University of Pennsylvania Perelman School of Medicine, Philadelphia, PA, United States

**Keywords:** primary open-angle glaucoma, mitochondrial genetics, African ancestry, neurodegeneration, perspective

## Abstract

Glaucoma, the leading cause of irreversible blindness worldwide, disproportionately affects individuals of African descent. Specifically, previous research has indicated that primary open-angle glaucoma (POAG), the most common form of disease, is more prevalent, severe, early-onset, and rapidly-progressive in populations of African ancestry. Recent studies have identified genetic variations that may contribute to the greater burden of disease in this population. In particular, mitochondrial genetics has emerged as a profoundly influential factor in multiple neurodegenerative diseases, including POAG. Several hypotheses explaining the underlying mechanisms of mitochondrial genetic contribution to disease progression have been proposed, including nuclear-mitochondrial gene mismatch. Exploring the fundamentals of mitochondrial genetics and disease pathways within the understudied African ancestry population can lead to groundbreaking advancements in the research and clinical understanding of POAG. This article discusses the currently known involvements of mitochondrial genetic factors in POAG, recent directions of study, and potential future prospects in mitochondrial genetic studies in individuals of African descent.

## 1 Introduction

Glaucoma is an ophthalmic disease that leads to progressive severe vision loss, accounting for the most common cause of irreversible blindness worldwide. Despite the robust and extensive literature currently covering this disease, there inevitably continues to be gaps in the depth and breadth of available research. This can be seen especially in areas surrounding underserved and overaffected populations, such as individuals of African descent. Large epidemiologic studies spanning multiple years have revealed the tangible health risks inherently associated with being Black, including but not limited to higher prevalence rates of systemic disease, premature onset of classically age-related conditions, unequal socioeconomic status, lower quality of life, and increased mortality risk (1–3). Despite these known associations with increased burden of disease as well as the resultant health and life consequences, individuals with African ancestry continue to be frequently underrepresented in research studies and trials (4). This can be seen across the spectrum of medical research and care, including in the field of ophthalmology (5).

In the few studies on primary open-angle glaucoma (POAG) that have centered around patients with African ancestry, unique aspects of disease etiology have been uncovered. These include earlier age of onset, more severe phenotypic presentations and outcomes, and more rapid disease progression (6, 7). Many underlying age-independent risk factors contributing to this accelerated disease process have been suggested, including vascular dysregulation, increased intraocular pressure (IOP), and thinner central corneal thickness (CCT) (8). Additionally, studies have identified differences in genetic risk for POAG between ancestry groups (9–12), which have become increasingly highlighted as more evidence surrounding genotype-phenotype connections emerge.

While nuclear DNA (nDNA) has traditionally remained in the forefront of general scientific interest, the implications of mitochondrial DNA (mtDNA) in disease processes has drawn growing attention in the research sphere (13). For POAG, findings from previous studies have suggested a role of mitochondrial dysfunction in the disease, such as the high energy requirement of retinal ganglion cells, phenotypic similarities to optic neuropathies caused by mtDNA mutations, polymorphisms in nuclear genes that encode mitochondrial proteins, and matrilineal bias in transmission (13–16). A combination of increased attention to mtDNA, desire for elucidation of the genetic contributors to POAG etiology, and call for enhanced research on under-represented ancestry groups has created a focal point of interest centered around mitochondrial genetic contributions to POAG in individuals of African descent. Several studies have been published in the recent decade reporting findings of distinctive mtDNA associations with POAG and African genetics (17–19). Through these studies, features of the mitochondrial genome have been reported in African patients as well as other communities around the world containing African lineages following the global African diaspora.

The discrepancy between novel findings identified in studies of individuals with African ancestry coupled with a pervasive disparity in baseline research foundations presents a thought-provoking point for discussion and potential for improvement. The objectives of this perspective article are to briefly summarize the background of POAG pathogenesis in individuals of African ancestry, highlight the current findings of POAG-associated mitochondrial genetics in this population, call attention to the need for research focused on understudied populations, and present future prospects in mitochondrial genetic studies on this disease in individuals of African descent.

## 2 Primary open-angle glaucoma

### 2.1 Disease background

POAG is estimated to affect 57.5 million individuals or 2.2% of the global population worldwide (20). This number is expected to grow according to general epidemiologic trends of an aging population and increased prevalence of risk factors contributory to POAG (20). Risk factors that carry well-established associations with the development and progression of POAG include increased IOP, older age, hypertension, obstructive sleep apnea syndrome, smoking, male sex, African ancestry, positive family history, and genetics (20). By 2040, 111.8 million individuals worldwide are estimated to be affected by POAG (20).

Patients can present at variable stages of the disease, from clinically asymptomatic to peripheral vision field defects, which can culminate in severe central vision loss. Because the disease is typically asymptomatic in early stages, up to half of patients with glaucoma remain undiagnosed, delaying treatment (21, 22). Currently, the only treatable component of disease is elevated IOP, and both medical and surgical therapeutic options are available. However, high IOP is neither necessary nor sufficient to develop POAG, as evidenced by a subset of POAG with no measured IOP elevation called normal tension glaucoma (23). Even in the setting of high IOP, IOP-lowering therapies have limited success with approximately 30% of patients continuing to experience disease progression despite adequate IOP control (24). Furthermore, existing therapies cannot regenerate retinal ganglion cells (RGCs), which are the primary cell type affected in POAG, or restore visual capacity (25). These observations demonstrate the presence of an unmet need for effective treatment modalities and management of POAG.

Regardless of the severity, the broad effects of the disease and consequential visual impairments present significant burdens on the health, personal, societal, and economic factors of the affected individual and society at large (26, 27). Significant impacts to quality of life are noted even during early stages of disease, with effects increasing as phenotypic severity progresses (26). The financial burden of disease manifests as both direct (e.g., medication, doctor visits, transportation) and indirect (e.g. lost productivity of patient and caregivers) costs to the patient and healthcare system. Financial costs have also been shown to directly relate to the duration, severity, and progression of the disease (26, 27). Additionally, these deleterious effects cannot be overlooked in asymptomatic undiagnosed patients, as delayed diagnosis often results in increased downstream consequences.

Regarding the background pathophysiology of glaucoma, many pathways remain ill-defined. POAG is characterized by progressive loss of RGCs, which are sensory neurons that collect all visual information from the eye, transmit these stimuli through axons that converge to form the optic nerve, and relay these signals to the brain for further processing. Loss of RGC’s results in structural defects of the optic nerve as well as function defects in signal transmission, causing specific, irreversible changes to the visual fields. POAG is a complex, multifactorial disease with many possible contributory underlying etiologies noted in the literature. Many different components of this disease have been proposed, including the mechanical theory of elevated IOP causing structural compression, variable IOP fluctuations throughout the day and night, and intraocular-intracranial pressure differences causing a stress-strain relationship across the lamina cribrosa (28–30). Another recently proposed disease mechanism includes the vascular theory that suggests underlying retinal vascular dysfunction predisposes the optic nerve to an insufficient blood supply, increasing vulnerability to neurodegeneration in the setting of additional stressors such as increased IOP (31).

### 2.2 Genetic background

POAG has a strong inherited component, but understanding of the disease’s genetics remains incomplete. Both nuclear and mitochondrial genes have been implicated in the many pathways contributing to POAG pathoetiology. Multiple polymorphisms in the *MYOC, OPA1, OPTN, TBK1, WDR36, SRBD1, ELOVL5, CAV1/CAV2*, and many other genes have been found to be associated with POAG (8, 32, 33). Suggested pathologic mechanisms resulting from these genetic alterations include induced RGC apoptosis and elevations in IOP (34). In the mitochondria, variants in the *MT-CYB, MT-ND4*, and *MT-ND5* genes as well as mitochondrial haplogroup K have also been associated with POAG (35, 36). These mitochondrial variants have been found to cause disturbances in lipid and carbohydrate metabolism, oxidative stress regulation, and overall mitochondrial integrity (34, 37). It is also important to note that the downstream effects of nuclear and mitochondrial genes are not mutually exclusive. Multiple previously identified nuclear genes related to the pathoetiology of POAG have been found to have a shared commonality of effect on mitochondrial structure and function (13).

Awareness of the impacts of mitochondrial genetic contributions to POAG pathogenesis has steadily grown in recent years as genome-wide association studies (GWAS) and other next-generation sequencing techniques provide molecular evidence of associations. A cohort study of patients with POAG that examined both nuclear and mitochondrial variations found potentially pathogenic mtDNA changes in approximately half of the cases but none of the control subjects (34). In particular, mitochondrial function has been heavily implicated in diseases of neurodegeneration, as the high energy requirements of neural tissues require optimization of mitochondrial homeostasis (38). A combination of nuclear genetics, mitochondrial genetics, and innate characteristics of neural tissue are all suggestive of the close relationship between the mitochondria and POAG pathogenesis.

Of note, many of these loci were identified in populations of European or Asian ancestry and have a reduced or unknown roles in individuals of African ancestry (9–12). For example, in five loci (*CDKN2B-AS1*, *TMCO1*, *CAV1*/*CAV2*, chromosome 8q22, *SIX1/SIX6)* that were previously identified to be associated with POAG in Caucasian populations on a genome-wide scale, only select single nucleotide polymorphisms (SNPs) in the *CDKN2B-AS1* and *SIX1/SIX6* loci were found to be associated with African American POAG samples (9). In this same study, no SNPs in any loci were significantly associated with Ghanaian POAG samples (9). A separate study of 15 SNPs of 15 loci (*CDC-TGRFB3, TMCO1, AFAP1, FOXC1, GMDS, CAV1/CAV2*, chromosome 8q22, *CDKN2B-AS1, ABCA1, ARHGEF12, ATXN2, SIX6, PMM2, GAS7, TXNRD2*) previously associated with POAG in European and Asian populations revealed no significant associations with Tanzanian, South African, or African American POAG samples (10). However, expanded analysis of SNPs in linkage disequilibrium demonstrated that the calculated combined genetic risk score of all 15 SNPs and *TXNRD2* (rs16984299) were significantly associated with the African descent POAG samples (10). Thus, inclusion and stratification of African-specific samples with consideration of genetic heterogeneity is necessary to provide a more accurate reflection the gene-disease associations in this population. **Table 1** provides a summary of genetic associations with POAG and the populations in which the association was discovered or replicated with genome-wide significance.

**Table:** Table 1. Genome-wide associations with POAG and populations of study.

Article	Populations	Ancestry	Genomic Regions	SNP	Allele	Association
Thorleifsson et al. (2010) (39)	European, Chinese	European, Asian	*CAV1/CAV2*	rs4236601	A	POAG
Burdon et al. (2011)(40)	Australian	European	*CDKN2B-AS1*	rs4977756	A	OAG
			*TMCO1*	rs4656461	G	OAG
Osman et al. (2012)(41)	Japanese	Asian	*CDKN2A-CDKN2B*	rs1063192	T	POAG
			*SIX6 *	rs10483727	T	POAG
Wiggs et al. (2012)(42)	Continental American	European	*CDKN2BAS*	rs2157719	G	POAG
			*SIX1/SIX6*	rs10483727	A	POAG
			*CDKN2BAS *	rs2157719	G	NPG
			Chromosome 8q22 probable regulatory region	rs284489	G	NPG
Liu et al. (2013) (9)	African American	African	*CDNK2B-AS1*	rs10120688	A	POAG
			*CDKN2B-AS1*	rs10965245	A	HPG
			*SIX1/SIX6*	rs11849906	G	NPG
Gharahkhani et al. (2014)(43)	Australian, American	European	*ABCA1*	rs2472493	G	POAG
			*AFAP1*	rs4619890	G	POAG
			*GMDS*	rs11969985	G	POAG
Chen et al. (2014)(44)	Chinese, Singaporean Chinese	Asian	*ABCA1*	rs2487032	A	POAG
			*PMM2*	rs3785176	G	POAG
Li et al (2015) (45)	Multiethnic Asian	Asian	*FNDC3B*	rs4894796	G	POAG
	European, Asian, American, Australian	Asian, African and European	*CDKN2B-AS1*	rs2157719	G	POAG
		Asian, African and European	*CDC7-TGFBR3*	rs1192415	G	POAG
Springelkamp et al. (2015) (46)	European, Australian	European	*ARHGEF12*	rs58073046	G	POAG
Bailey et al. (2016)(47)	Australian, American, European, Singaporean Chinese	European, Asian	*TXNRD2*	rs35934224	T	POAG
			*ATXN2*	rs7137828	T	POAG
			*FOXC1*	rs2745572	A	POAG
Bonnemaijer et al. (2018) (10)	African, African American	African	*TXNRD2*	rs16984299	C	POAG
Gharahkhani et al. (2021) (11)	Multiethnic multiancestral	African, European, Asian	127 loci	–	–	POAG
	African, African American	African subgroup	*IQGAP1*	rs16944405	A	POAG
Mitochondrial DNA						
Banerjee et al. (2013) (35)	Eastern Indian	Eurasian	*MT-ND5*	–	–	POAG
Collins et al. (2016)(17)	African American	African	Haplogroups L1c2, L1c2b, and L2	–	–	POAG
Collins et al. (2018)(19)	African American	African	*MT-CO1*	rs879053914	A	POAG
Gudiseva et al. (2019) (18)	African American	African	Haplogroups non-L3 (L0, L1, L2, and non-L)	–	–	POAG
Lo Faro et al. (2021)(36)	Dutch	European	*MT-ND4*	rs2853496	A	POAG
			*MT-CYB*	rs35788393	T	POAG
			Haplogroup K	–	–	POAG

POAG, primary open-angle glaucoma; OAG, open-angle glaucoma; HPG, high-pressure glaucoma; NPG, normal-pressure glaucoma.

## 3 Primary open-angle glaucoma in the African ancestry population

### 3.1 Overaffected and understudied

The United States consistently leads in scientific research funding, output, and clinical trials (48, 49). The United States is also home to the second-largest (following Brazil) African diaspora population with 47.2 million individuals, or approximately 14.2% of the nation’s population (50, 51). Yet, there remains a disparity in the extent to which African Americans are included and studied in the scientific research of the nation. Research within this specific population of African descendants also holds immense value due to their highly heterogeneous nature stemming from a complex history of African origins coupled with early admixture with European and American populations (52). This unique genetic diversity not only reflects the rich tapestry of African American heritage but also presents a compelling opportunity for scientific exploration in a research area with demonstrated need.

The impacts of POAG are particularly pronounced within populations of African ancestry, who are over-affected both in disease and health-related quality of life. In large epidemiologic studies, prevalence of POAG was found to be four to six times higher in African Americans (4-8%) compared to European Americans (1-2%) with a six to eight times higher prevalence of glaucoma-related blindness (8, 53, 54). Onset of the disease is also estimated to be approximately 10 years earlier in Black individuals compared to White individuals (55). In studies of patients in Ghana, the prevalence of POAG at 30 years old was 6%, and 21% of patients were 10-39 years old (56). These findings are in contrast to the average onset of glaucoma beginning at 40 years old in Caucasian patients (57). Glaucoma in the African descent population is also associated with more severe phenotypes with poorer outcomes (6, 7). Damage to the disc, retina, and visual field both at initial diagnosis and progression with disease were more significant in individuals of African descent (55, 56, 58). With the same elevated mean IOP levels and similar access to care, African Americans were also more likely to present with more severe visual field defects than European Americans (6). African American individuals were also found to have more predisposing risk factors, including thinner central corneas, elevated IOP’s, and increased oxidative stress levels (8, 59, 60).

Despite increased severity at all stages of disease, individuals of African descent remain underdiagnosed and undertreated for POAG compared to their counterparts of the same national healthcare systems. African Americans presented to healthcare services later and have an estimated 45% lower than expected rate of glaucoma surgery (7, 54). In a large United States national study utilizing Medicare data, African American patients recorded lower utilization of outpatient visits and testing, but higher inpatient and emergency department encounters, a result which persisted following stratification by socioeconomic status (61). The racial disparities in health outcomes contribute to the aforementioned downstream burdens on quality of life.

This discrepancy between the population affected compared to the population treated also applies to the realm of research studies. Many of the background literature research that informs diagnostic and therapeutic recommendations are lacking in adequate representation from all affected race and ethnic groups. In a retrospective review of United States clinical trials over the last two decades, the authors found that minority race or ethnic group enrollment proportions were below that of population census estimates (62). They also noted a wide heterogeneity in the practice of enrollment and reporting across the clinical trials. Twenty-one percent of trials reported no Black participants, fewer than 44% of trials reported any data regarding race or ethnicity, and fewer than 25% of trials reported data encompassing all five major race and ethnicity groups (62). Notably, this study also demonstrated that industry and academic funding were negatively correlated with race or ethnicity reporting (62). In a study of ophthalmology drug clinical trials specifically, the same finding of underrepresentation in the expected distribution of minority racial and ethnic groups was demonstrated (5).

Individuals of African descent face a disproportionately higher risk of POAG onset, progression, severity, and health-related quality of life burden (6–8, 53–56, 58). Yet, this same population is often underrepresented in research studies aimed at understanding and addressing this disease and related health conditions. This double burden of being overaffected and understudied underscores the urgent need for increased research and awareness to mitigate the impact of glaucoma within this vulnerable population as well as improve overall understanding of the disease.

### 3.2 Key mitochondrial genetic findings

As society at large continually seeks to improve diversity, equity, and inclusion in all aspects of human interaction, the scientific community is also called upon to identify and address areas of improvement. Enhancing the representation of minority groups is a critical next step to elevating the ethical standards of research and science. In addition to this inherent value, exploring mitochondrial genetic variation in African ancestry individuals has the potential to offer unique insights due to the specific characteristics of disease and genetics in this population. This direction of study holds tremendous potential for elucidating the mechanisms of underlying disease pathogenesis, as well as identifying novel, precise therapeutic targets.

Populations of African descent are more genetically diverse than those of non-African descent (63). Many historical and environmental factors contribute to this diversity, including demographic trends, migratory patterns, external pressures, disease exposure, and natural selection (63). Phylogenetic analyses of mtDNA haplogroups have localized the oldest mtDNA lineages to African populations, with all humans having a maternal ancestry tracing back to a single African mtDNA from 194,300 +/- 32,500 years ago (64). According to this finding, all human mitochondrial genetic material is originally derived from distant African ancestry. This shared genetic thread offers profound insights into a collective biologic history. The study of African mitochondrial DNA samples holds great value to enhance understanding of disease as well as unlock novel targets for therapy. Thus, this past connection between mitochondrial genetics and African heritage has potential implications for present findings and future applications in all individuals. As variants in mtDNA arose sequentially over subsequent years, new distinct haplogroups sharing a common lineage with the same genetic variants emerged, ultimately leading to modern European and Asian mtDNA haplotypes (30, 63, 65). In glaucoma, this genetic diversity can be seen in the difference in prevalence rates amongst various communities of African ancestry, with estimated prevalences of 1% in Nigeria, 8% in Ghana, 7-9% in African-Caribbeans, and 4% in African-Americans (8).

The heterogeneity of African American genomes in particular, containing both African origin and non-African branches of mitochondrial lineages, creates unique opportunities for study within the same sample. In genetic studies specific to African American populations, researchers have found increased association of African mtDNA haplogroups L1c2, L1c2b, and L2 in African American patients with POAG (17). It is estimated that approximately 25% of African Americans carry these haplogroups, contributing to the population’s increased risk of POAG (17). These findings were demonstrated in another study that showed certain African non-L3 mitochondrial haplogroups (L0, L1, L2, and non-L0) were associated with higher POAG risk in males compared to the L3 haplogroup, which serves as the major branching point for contemporary non-African lineages (18). Additionally, the *MT-CO1 V83I* polymorphism, which is found to be part of the previously mentioned African haplogroups, was also found independently to be strongly associated with POAG in African American males (19). Mitochondrial genetic studies in the African American population in relation to POAG are only now beginning to emerge (17–19). These initial findings of association provide motivation for continued investigation into the connections between African American mitochondrial genetics and diseases of interest.

## 4 Challenges and future directions

Both social and scientific challenges contribute to the difficult task of conducting genetic studies in the African American population. Recruitment in clinical trials and basic science studies have been affected by economic concerns, barriers to transportation access, limited exposure to research, low education levels, mistrust in the healthcare system, historical encounters with inequities, and investigator bias (4). The Primary Open-Angle African American Glaucoma Genetics (POAAGG) study successfully recruited over 10,200 individuals of African descent over a nine year period in the city of Philadelphia to participate in genetic sampling. Interviews of individuals participating in this study revealed common themes influencing decisions to enroll. These include a desire to know more about personal health, opportunity to contribute to the health of others, and exposure to the study through a respected source in their community (66). About one out of three of these respondents mentioned past and current racial discrimination in medical research contributing to their decision-making process (66). This study reflected many of the social, environmental, and historical considerations in the African American community when choosing to participate in a medical research study. These concerns are further magnified when the research involves genetic sampling and storage of DNA samples. Future studies recruiting African Americans can utilize targeted strategies to enhance enrollment results. These include trusted collaborations with respected community partners, combined enrollment with health care services, offers of economic support for participation, and emphasizing potential to give back to the community by improving health outcomes for future generations.

The admixed population and high heterogeneity of genetic material in individuals of African descent provides interesting opportunities for exploration. However, these also present distinct challenges and higher requirements for research studies. High genetic diversity results in less likelihood of reproducibility of results, especially across institutions in different locales exposed to different communities of African descent. This requires high demands on sample size across locations (10). Even within a single subgroup, increased genetic admixture requires extensive genetic background data. African American mitochondrial data often traces back to multiple African ethnic groups rather than a single group or locale of ancestry. Only a small fraction of the approximately 2000 African ethnic groups, especially sub-Saharan groups, have been sampled for large-scale genetic studies of genome-wide variations (63). In order for future studies to assess study samples for variants and polymorphisms of interest, representative data of the African gene pool needs to be established to use as a comparison frame of reference.

Advancements are currently underway to expand the presence of African populations in research studies across all disciplines. Improvements have been made in enrollment of minority race and ethnic group individuals in U.S. clinical trials over the past decades although they are still underrepresented (62). Large-sample African American genetic studies are beginning to emerge, such as the POAAGG study containing 10,255 participants at the time of writing (67).

## 5 Discussion

Research in African ancestry individuals presents a unique dichotomy between discovery and retroaction. While many exciting advances are being made, there are still prior gaps that require amendment. Acknowledgement of this discrepancy can help fuel discussions surrounding directions for improvement. Improved sampling and databases across all locales are needed to inform future studies of pathogenicity. The current direction of research is moving towards increased awareness regarding the need and value of African genetic study that is coupled with more advanced and widely available genetic testing techniques. Residing at the intersection point of these multiple general trends in the current sphere of research, the future of African American mitochondrial genetics in the study of POAG remains bright.

## Data availability statement

The original contributions presented in the study are included in the article/supplementary material. Further inquiries can be directed to the corresponding author.

## Author contributions

GK: Conceptualization, Writing – original draft. RS: Writing – review & editing. JO: Funding acquisition, Supervision, Writing – review & editing.
